# Lower Expression of miR-26a in PBMCs Indicates the Occurrence of Early-Onset Neonatal Sepsis and Is Partly Mediated by the Upregulation of PTEN

**DOI:** 10.3389/fped.2021.678205

**Published:** 2021-08-24

**Authors:** Ziyan Zhao, Jiajie Zhang

**Affiliations:** Department of Pediatrics, Henan Provincial People's Hospital, People's Hospital of Zhengzhou University, Zhengzhou, China

**Keywords:** biomarkers, early-onset neonatal sepsis, microRNA-26a, peripheral blood mononuclear cells, phosphatase and tensin homolog

## Abstract

**Aim:** It is difficult to identify neonatal sepsis early due to the lack of specific markers. The aim of the present study was to explore whether miR-26a expression in peripheral blood mononuclear cells (PBMCs) could be used as a diagnostic marker of the disease and whether phosphatase and tensin homolog (PTEN) was involved in suppressing miR-26a expression.

**Methods:** A total of 51 early-onset septic newborns and 102 healthy newborns were included. Blood specimens were collected from septic newborns at the time of clinical diagnosis (baseline) and again between 72 and 96 h after birth. Blood specimens were collected from healthy newborns on admission. The expressions of miR-26a and PTEN in PBMCs were measured using real-time quantitative PCR (RT-qPCR). Other data, including hemoculture, were collected from medical records.

**Results:** In septic newborns with and without a positive hemoculture, a lower baseline level of miR-26a in PBMCs was associated with a higher risk of disease. Additionally, at baseline, there was a certain linear relationship between the levels of miR-26a and two serological inflammatory markers (i.e., white blood cell count and C-reactive protein level) in septic newborns. In addition, the baseline expressions of miR-26a and PTEN showed a reverse linear relationship. Compared with those at baseline, the expression of miR-26a was higher and the expression of PTEN was lower in septic newborns starting at 72 h after birth.

**Conclusion:** A lower baseline miR-26a expression in PBMCs indicated the occurrence of early-onset neonatal sepsis, and a reduced miR-26a expression might be partly related to the inflammatory process and PTEN upregulation.

## Introduction

Due to their immature immune system, newborns are easily affected by infectious diseases. Among them, neonatal sepsis is the most severe, with a high mortality. At present, neonatal sepsis is usually divided into two clinical types: early-onset sepsis (EOS) and late-onset sepsis (LOS). The former refers to a disease that occurs during the first 72 h of life; the latter indicates a process that develops after the first 72 h (1, 2). The two types have different pathogens, risk factors, and pathogenesis.

Early diagnosis and treatment are important for improving the outcomes of neonatal sepsis. However, the disease is confirmed by microbiological blood culture, which takes days and probably has a high rate of false-positive results. In this context, doctors are usually dependent on the clinical manifestations, white blood cell count, and other inflammatory markers (such as C-reactive protein) to establish an initial diagnosis and provide empirical antibiotic therapy (1, 2). This is subjective and prone to misdiagnoses. Therefore, a novel biochemical marker is urgently needed to compensate for the deficiencies in the current diagnostic process.

Peripheral blood mononuclear cells (PBMCs) are currently regarded as useful carriers of bio-information. Their gene expression profiles have significant diagnostic and prognostic significance in malignant tumors and obesity (3, 4). Bellaver et al. explored the potential effect of PBMCs on septic encephalopathy and confirmed that PBMCs are involved in the regulation of astrocyte reactivity (5). Therefore, we have reasons to believe that PBMCs play a certain role in sepsis.

MicroRNAs (miRNAs) are single-stranded non-coding nucleic acid molecules containing 19–22 nucleotides (6). They bind to specific genes and regulate their expression, resulting in the promotion or inhibition of physiological activities. MicroRNA-26 (miR-26) is an important miRNA that exerts a series of biological functions in malignant tumors and cardiovascular diseases (7–9). More importantly, one previous study explored the regulatory role of miR-26a in neonatal sepsis and reported that the expression of miR-26a is significantly decreased in neonates with sepsis (10). This is an interesting but a preliminary study, and the clinical significance of miR-26a in neonatal sepsis is still not fully understood.

In addition, the downstream targets of miR-26a in neonatal sepsis are also unknown. Some studies have reported that phosphatase and tensin homolog (PTEN) is a molecular target of miR-26a (11, 12), and PTEN/PI3K/AKT signaling is involved in the regulation of sepsis (13, 14). However, to date, the miR-26a/PTEN/PI3K/AKT axis has not been explored in neonatal sepsis.

Taken together, we speculated that the expression of miR-26a in PBMCs predicts the occurrence of EOS. Therefore, we conducted an observational study that included 51 Chinese newborns with EOS to test this hypothesis and to begin to explore the potential mechanisms involved.

## Materials and Methods

This study was approved by the Ethics Committee of Henan Provincial People's Hospital. Parents of the newborns agreed to participate in the study and signed a written informed consent form.

### Newborns

A total of 51 Chinese newborns with EOS were prospectively and continuously enrolled from the Department of Pediatrics, Henan Provincial People's Hospital, between January 2016 and July 2020. The inclusion criteria were as follows: (1) presented with clinical features of sepsis during the first 72 h of life. The clinical features affected at least three distinct systems, and possible clinical features included difficulty breathing, cyanosis, tachycardia, bradycardia, irritability, lethargy, food intolerance, unexplained jaundice, body temperature instability, and petechiae. (2) Increased white blood cell count and C-reactive protein levels in the serum; (3) positive or negative microbiological blood culture results; and (4) the presence or absence of premature delivery (1). Based on the above criteria, three pediatricians made the diagnosis by consensus.

During the same period, 102 healthy newborns, from the Department of Obstetrics, Henan Provincial People's Hospital, were included and served as controls.

### Blood Collection

Two peripheral blood specimens (~1–2 ml) were collected from each septic newborn, once at baseline (at the time of diagnosis) and again between 72 and 96 h after birth. Peripheral blood specimens were collected from each healthy newborn on admission.

All specimens were inoculated into aerobic bottles with pediatric hemoculture medium (BIOSCAN, Saddle Brook, NJ, USA) and incubated with agitation at 37°C for 4 h. Afterwards, the PBMCs were isolated using OptiPrep density gradient separation (Sigma-Aldrich, St. Louis, MO, USA) according to the manufacturer's instructions.

### Real-Time Quantitative Polymerase Chain Reaction

Total RNA extraction was conducted with a commercial TRIzol® reagent kit following the manufacturer's protocol (Invitrogen, Waltham, MA, USA). Afterwards, the concentration of the RNA was quantified using a NanoDrop 2000 spectrophotometer (Thermo Scientific, Waltham, MA, USA).

To measure the expression of miR-26a, a total of 100 ng of total RNA was used to synthesize complementary DNA (cDNA) templates using a TaqMan® miRNA reverse transcription kit (Applied Biosystems, Waltham, MA, USA). The expression of miR-26a was quantified with a TaqMan MicroRNA Assay Kit and TaqMan Master Mix (Applied Biosystems). The analysis was conducted with primers for miR-26a (assay ID 000405) and the internal reference U6 (assay ID 001973).

To measure the messenger RNA (mRNA) expressions of PTEN, PI3K, and AKT, 1 μg of total RNA was reverse transcribed by a high-capacity cDNA reverse transcription kit for mRNA (Applied Biosystems), and the quantitative process was performed with SYBR Green PCR Master Mix (Applied Biosystems) according to the manufacturer's instructions. GAPDH served as the internal reference. The primer sequences were as follows: the PTEN forward sequence was 5′-GGACGAACTGGTGTAATGATATG-3′ and the reverse sequence was 5′-TCTACTGTTTTTGTGAAGTACAGC-3′; the PI3K forward sequence was 5′-ATCGACAAGCGCATGAACAGC-3′ and the reverse sequence was 5′-TACCACGGAGCAGGCGTAGCAG-3′; the AKT forward sequence was 5′-TGAGCGACGTGGCTATTG-3′ and the reverse sequence was 5′-CAGTCTGGATGGCGGTTG-3′; the GAPDH forward sequence was 5′-GGGAAACTGCGGCGTGAT-3′ and the reverse sequence was 5′-AAAGGTGGAGGAGTGGGT-3′.

All the reactions were carried out in a 7900HT Fast Real-Time PCR System (Applied Biosystems). The reaction conditions for miR-26a were 90°C for 60 s, 95°C for 15 s, and 60°C for 30 s, for a total of 40 cycles. The reaction conditions for PTEN/PI3K/AKT mRNA were 95°C for 30 s, 95°C for 5 s, and 57°C for 30 s, for a total of 45 cycles. All experiments were repeated three times, and the relative expression levels were calculated using the 2^−Δ*ΔCt*^ method.

### Information Collection

The weight, temperature, heart rate, respiratory rate, and serological markers (i.e., white blood cell count and C-reactive protein) at the time of diagnosis were collected from medical records. Sex, premature delivery, prognosis, and other necessary information were also obtained from medical records. The information collection process was performed by two well-trained researchers using a predefined table.

### Statistical Analysis

Continuous variables are expressed as the mean ± standard deviation. Differences between two variables were determined using independent samples *t*-tests. Pearson's correlation analysis was adopted to identify linear relationships between two variables. A *P* < 0.05 was regarded as statistically significant.

Categorical variables are shown as frequencies and constituent ratios. Differences between two variables were determined using the chi-square test. If the *P* < 0.05, it was considered statistically significant.

Multivariate logistic regression analysis was used to determine the relationship between miR-26a expression in PBMCs and the occurrence of EOS in newborns. All newborns with EOS and controls were included in the analysis. Several confounding factors (i.e., age, weight, temperature, heart rate, respiratory rate, and premature delivery) were adjusted for to avoid the potential effects of these factors on the results. Odds ratios (ORs) and 95% confidence intervals (95% CIs) were reported. If a 95% CI did not include the value 1, then there was a statistically significant difference.

Multivariate Cox regression analysis was adopted to explore the relationship between miR-26a expression in PBMCs and the prognosis (i.e., septic death) of the newborns. All patients, both surviving and non-surviving, were included. After adjusting for confounding factors, hazard ratios (HRs) and 95% CIs were reported. If a 95% CI did not include the value of 1, it was regarded as statistically significant.

All analyses were performed using SPSS 17.0 (Chicago, IL, USA).

## Results

### Baseline Characteristics and the Prognosis of the Newborns

As shown in Table 1, there were 51 newborns with EOS (the total EOS group) and 102 healthy newborns (the control group). Among the newborns with EOS, 32 tested positive for hemoculture (EOS_POS group); those with negative hemoculture (*n* = 19) were diagnosed based on the clinical features, white blood cell count, and serum levels of C-reactive protein (EOS_NEG group).

**Table 1:** Characteristics of newborns with early-onset neonatal sepsis.

	**Total EOS^a ^**	**Control**	***P*^b^**	**EOS_POS^a^**	**EOS_NEG^a^**	***P*^b^**
Total (*n*, %)	51 (100.0)	102 (100.0)	—	32 (100.0)	19 (100.0)	—
**Baseline**						
Male (*n*, %)	27 (52.9)	56 (54.9)	0.818	18 (56.3)	9 (47.4)	0.539
Body weight (kg)	2.7 ± 0.7	3.2 ± 0.5	<0.001	2.8 ± 0.8	2.6 ± 0.7	0.224
Temperature (°C)	37.3 ± 1.7	36.5 ± 1.4	0.003	37.2 ± 1.8	37.4 ± 1.6	0.681
Heart rate (beats/min)	149.5 ± 15.5	143.6 ± 11.9	0.011	149.1 ± 16.1	150.1 ± 14.9	0.834
Respiratory rate (breaths/min)	55.5 ± 6.8	50.6 ± 7.9	<0.001	56.2 ± 6.4	54.3 ± 7.4	0.347
White blood cell count (×10^9^)	31.9 ± 6.4	16.0 ± 3.3	<0.001	31.3 ± 6.1	32.8 ± 7.0	0.423
C-reactive protein (mg/l)	69.4 ± 29.5	5.1 ± 3.0	<0.001	67.8 ± 29.1	72.3 ± 30.8	0.604
Premature delivery (*n*, %)	20 (39.2)	9 (8.8)	<0.001	13 (40.6)	7 (36.8)	0.789
**Prognosis**						
Death (*n*, %)	6 (11.8)	0 (0.0)	—	6 (18.8)	0 (0.0)	—

a *“Total EOS”, “Newborns with clinical features of sepsis”; “EOS_POS”, “Newborns with positive haemoculture”; “EOS_NEG”, “Newborns with negative haemoculture.”*

b *Difference of continuous variables was detected using independent t sample test, and difference of categorical variables was detected using chi-square test. “P **<** 0.05” indicated statistically significant difference*.

The temperature, heart rate, respiratory rate, white blood cell count, C-reactive protein, and premature delivery rate were significantly higher in the total EOS group than those in the control group (*P* = 0.003, *P* = 0.011, *P* < 0.001, *P* < 0.001, *P* < 0.001, and *P* < 0.001, respectively), and the body weight was lower in the total EOS group compared with the control group (*P* < 0.001).

All the markers mentioned above (i.e., body weight, temperature, heart rate, respiratory rate, white blood cell count, C-reactive protein, and premature delivery rate) were equally distributed between the EOS_POS group and the EOS_NEG group (*P* = 0.224, *P* = 0.681, *P* = 0.834, *P* = 0.347, *P* = 0.423, *P* = 0.604, and *P* = 0.789, respectively).

There were six deaths, and all of them were in the EOS_POS group.

### Baseline Expression of miR-26a in PBMCs

**Figure 1:**
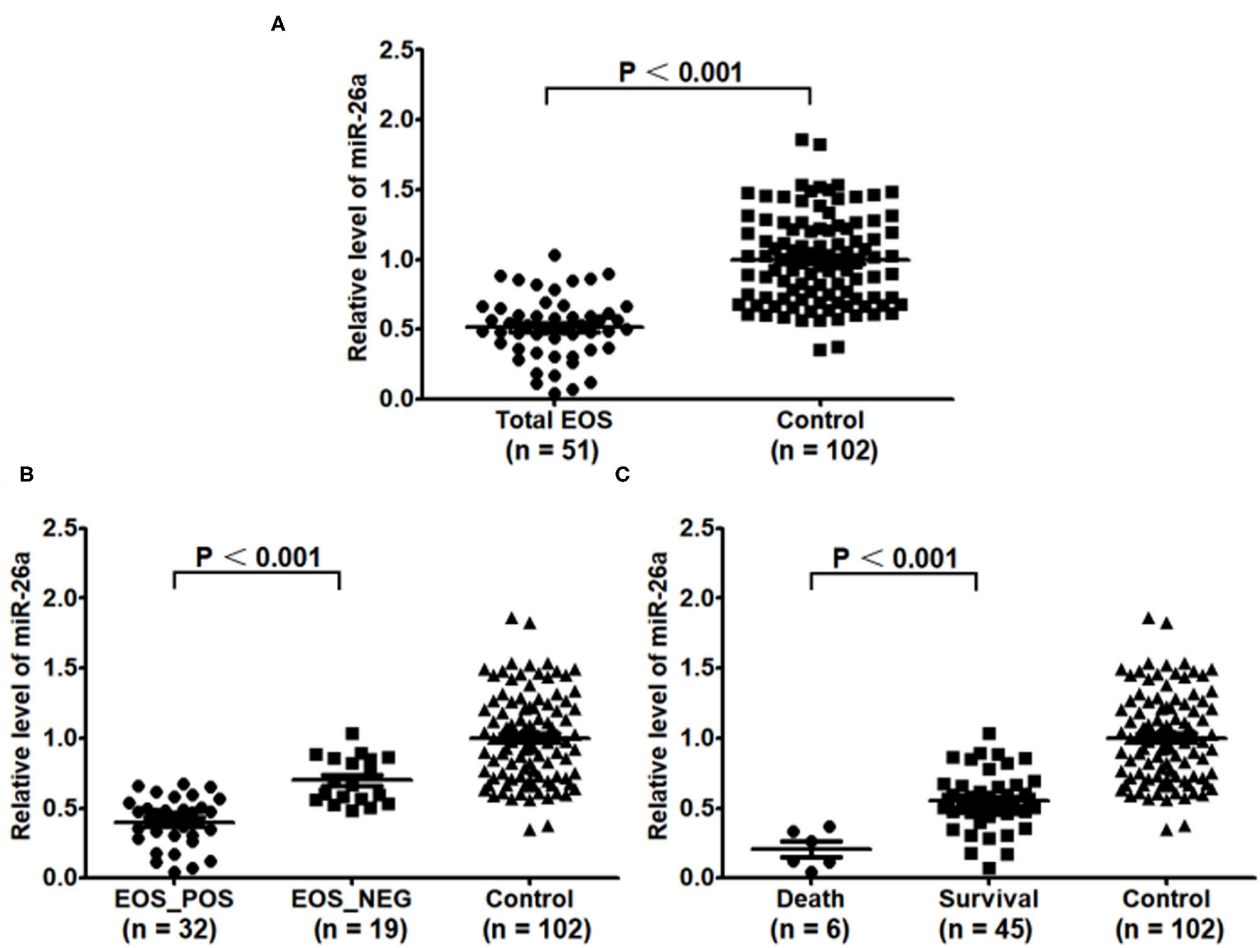
Baseline expression of miR-26a in peripheral blood mononuclear cells. **(A)** Baseline expression of miR-26a in the EOS newborns and control newborns was compared. **(B)** Baseline expression of miR-26a in the newborns with positive hemoculture and with negative hemoculture was compared. **(C)** Baseline expression of miR-26a in the dead newborns and living newborns was compared. In the process, independent sample t-test was adopted, and “*P* < 0.05” indicated a statistically significant difference. Total EOS, Newborns with clinical features of sepsis; EOS_POS, Newborns with positive hemoculture; EOS_NEG, Newborns with negative hemoculture.

Figure 1 shows that the expression of miR-26a was significantly decreased in the total EOS group compared with the control group (*P* < 0.001). Additionally, the expression of miR-26a was decreased in the EOS_POS group compared with the EOS_NEG group (*P* < 0.001) and was lower in the newborns who died than in those surviving (*P* < 0.001).

### Association of Baseline miR-26a With EOS Development and Death

As shown in Table 2, the data suggested that a lower expression of miR-26a in PBMCs was associated with a higher risk of EOS in newborns (OR = 2.29, 95% CI = 1.67–3.16). The analysis was performed again to assess the association of miR-26a with the occurrence of EOS separately with positive and negative hemoculture; a significant relationship between miR-26a expression and disease was still confirmed (OR = 4.35, 95% CI = 2.18–8.68; OR = 1.63, 95% CI = 1.16–2.31).

**Table 2:** Association of baseline miR-26a in peripheral blood mononuclear cells with early-onset neonatal sepsis or death.

	**Univariate OR (95%CI)^a^^,^^b^**	**Multivariate OR (95%CI)^a^^,^^b^**
**Neonatal sepsis patients and controls**		
Lower miR-26a and Total EOS	2.10 (1.63–2.71)	2.29 (1.67–3.16)
Lower miR-26a and EOS_POS	3.67 (2.10–6.39)	4.35 (2.18–8.68)
Lower miR-26a and EOS_NEG	1.62 (1.25–2.09)	1.63 (1.16–2.31)
	**Univariate HR (95%CI)** ^a^ ^,^ ^b^	**Multivariate HR (95%CI)** ^a^ ^,^ ^b^
**Survival and dead patients**		
Lower miR-26a and Death	1.06 (0.92–1.21)	1.06 (0.92–1.22)

a *“Total EOS”, “Newborns with clinical features of sepsis”; “EOS_POS”, “Newborns with positive haemoculture”; “EOS_NEG”, “Newborns with negative haemoculture”; OR, Odds ratio; HR, Hazard ratio; 95% CI, 95% Confidence interval*.

b *Association of miR-26a with neonatal sepsis was detected using univariate and multivariate logistic regression analysis. Association of miR-26a with death was detected using univariate and multivariate COX regression analysis. Multivariate model was adjusted by gender, weight, temperature, heart rate, respiratory rate, and premature delivery*.

However, the study did not find a significant relationship between miR-26a expression in PBMCs and septic death in newborns (HR = 1.06, 95% CI = 0.92–1.22).

### Baseline Relationship Between miR-26a and Inflammatory Markers

**Figure 2:**
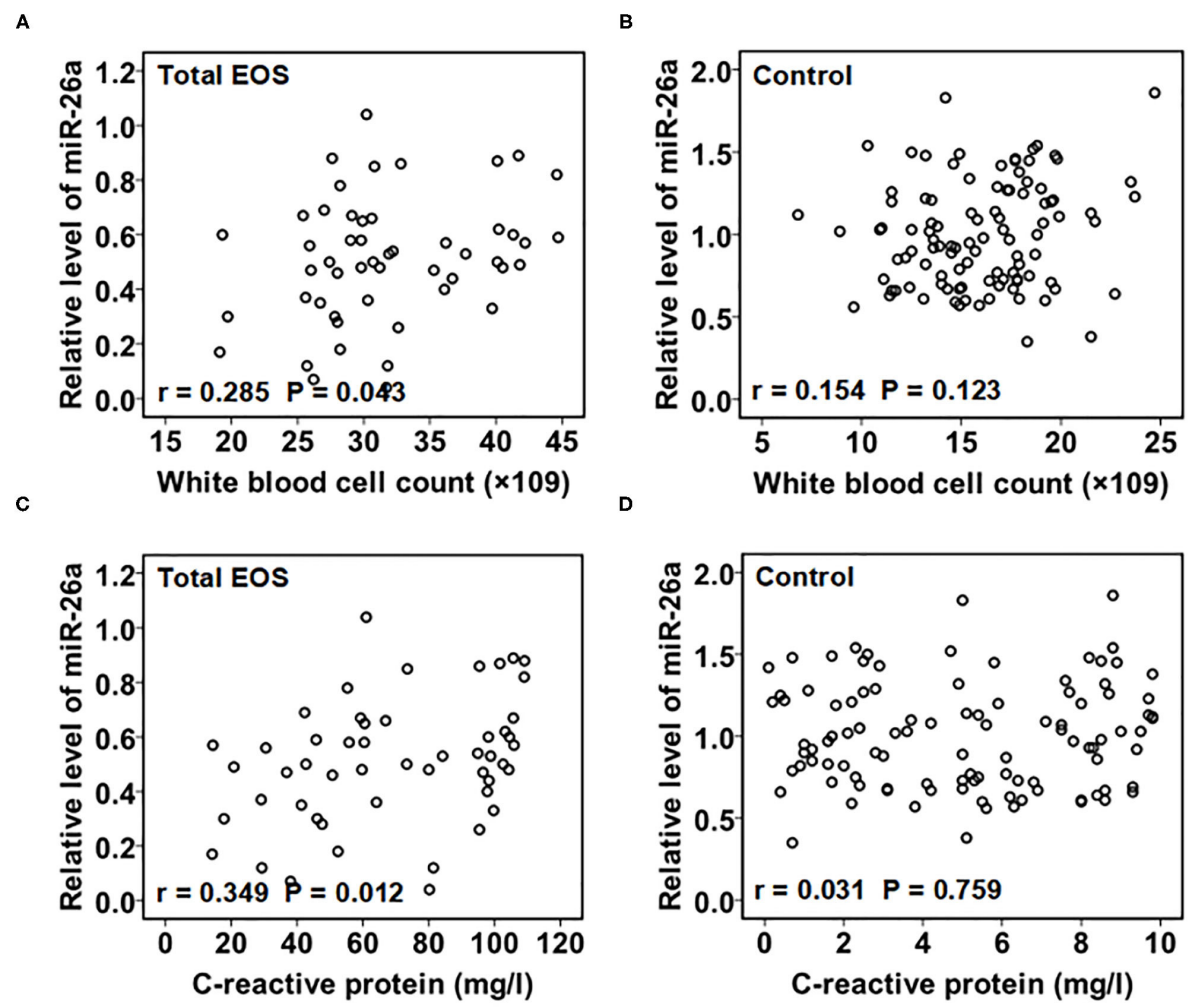
Association of baseline miR-26a in peripheral blood mononuclear cells with baseline C-reactive protein and white blood cell count. In **(A,B)**, baseline association between miR-26a and white blood cell count was assessed separately in the total EOS and control groups. In **(C,D)**, baseline association between miR-26a and C-reactive protein was assessed separately in the total EOS and control groups. In the process, Pearson correlation analysis was adopted, and “*P* < 0.05” indicates a statistically significant difference. Total EOS, Newborns with clinical features of sepsis.

As shown in Figure 2, there was a linear relationship between miR-26a expression in PBMCs and the white blood cell count in the serum in the total EOS group (*r* = 0.285, *P* = 0.043). A linear relationship between the expression of miR-26a in PBMCs and the serum C-reactive protein levels in the total EOS group was also found (*r* = 0.349, *P* = 0.012). In the control group, the expression of miR-26a in PBMCs did not have a significant association with the serum white blood cell count or the C-reactive protein level (*r* = 0.154, *P* = 0.123; *r* = 0.031, *P* = 0.759).

### Differences in miR-26a Levels Between Baseline and 72 h After Birth

**Figure 3:**
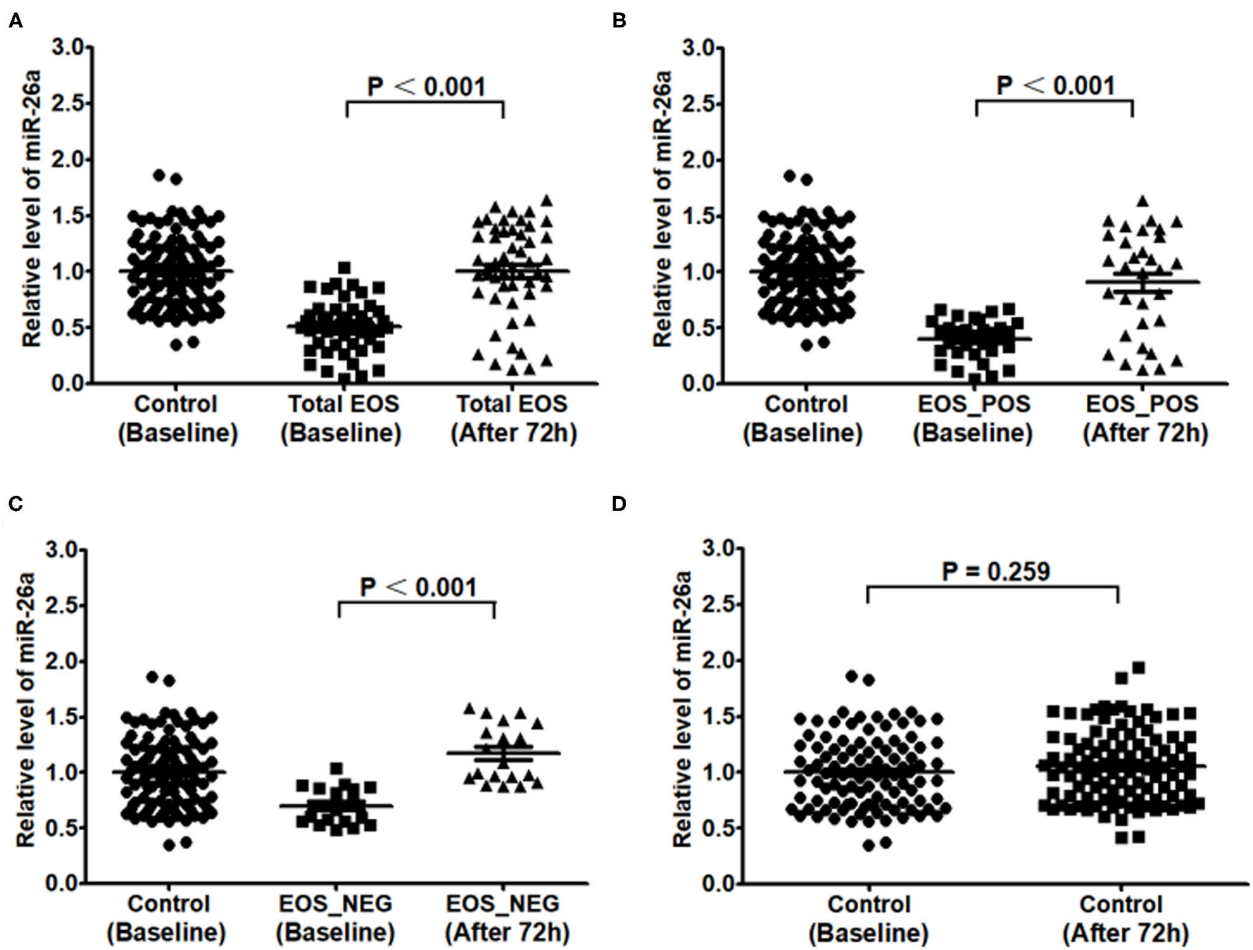
Expression of miR-26a in peripheral blood mononuclear cells at baseline and at 72 hours after birth. In **(A–D)**, expression of miR-26a at baseline and after 72 hours was compared separately in the total EOS group, EOS_POS group, EOS_NEG group and control group. In the process, independent sample *t*-test was adopted, and “*P* < 0.05” indicates a statistically significant difference. Total EOS, Newborns with clinical features of sepsis; EOS_POS, Newborns with positive hemoculture; EOS_NEG, Newborns with negative hemoculture.

As shown in Figure 3, the expression of miR-26a in PBMCs after the first 72 h was higher than that at baseline in the total EOS group, EOS_POS group, and EOS_NEG group (*P* < 0.001, *P* < 0.001, and *P* < 0.001, respectively). However, in the control group, there was no evidence of the expression of miR-26a changing over time (*P* = 0.259).

### Expression of PTEN in PBMCs

**Figure 4:**
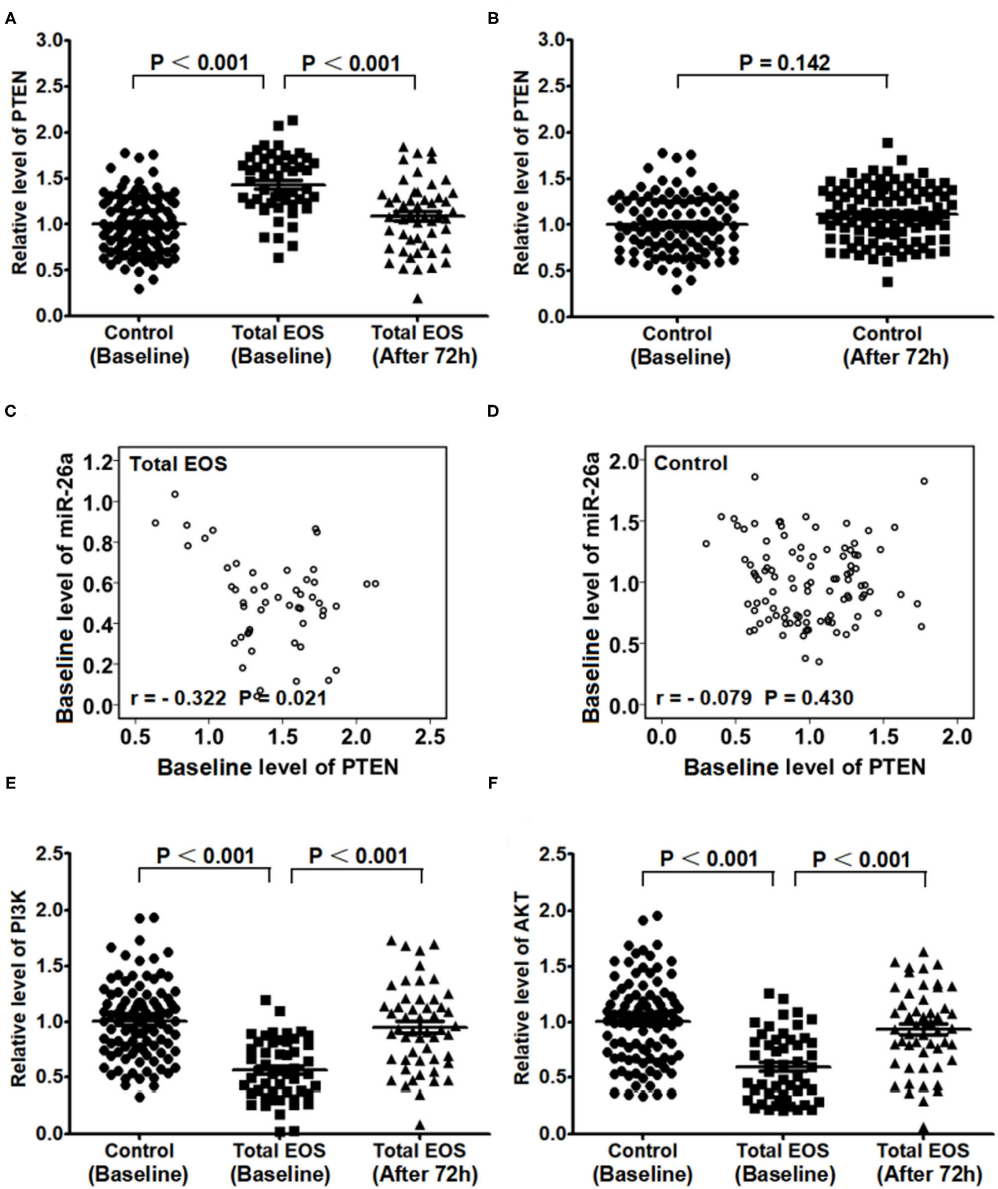
Expression of PTEN/PI3K/AKT in peripheral blood mononuclear cells at baseline and 72 hours after birth. **(A)** Baseline expression of PTEN in the control and total EOS groups was compared. In **(A,B)** Expression of PTEN at baseline and after 72 hours was compared separately in the total EOS group and control group. In **(C,D)** Baseline association between miR-26a and PTEN was assessed separately in the total EOS group and control group. In **(E,F)**, baseline expression of PI3K and AKT was compared between the control group and total EOS group, and expression of the proteins at baseline and after 72 hours was compared in the total EOS group. Differences in continuous variables were detected using independent sample t-tests. The association of miR-26a with PTEN was detected using Pearson correlation analysis. “*P* < 0.05” indicates a statistically significant difference. Total EOS, Newborns with clinical features of sepsis.

As shown in Figure 4A, the baseline expression of PTEN in PBMCs was higher in the total EOS group than that in the control group (*P* < 0.001). From baseline to 72 h after birth, the expression of PTEN in the total EOS group was significantly decreased (*P* < 0.001). Figure 4B shows that there was no difference in PTEN expression between the two time points mentioned above in the control group (*P* = 0.142).

As shown in Figures 4C,D, there was a significant linear relationship between the baseline levels of miR-26a and PTEN in the total EOS group (*r* = −0.322, *P* = 0.021), but not in the control group (*r* = −0.079, *P* = 0.430).

Figures 4E,F show the opposite trend for PTEN. The baseline expressions of PI3K and AKT in PBMCs were lower in the total EOS group than those in the control group (*P* < 0.001 and *P* < 0.001, respectively). During the first 72 h after birth, the expressions of these two genes were significantly increased in the total EOS group (*P* < 0.001 and *P* < 0.001, respectively).

## Discussion

A published study focusing on neonatal sepsis in 2018 suggested that the expression of IL-6 was significantly upregulated in PBMCs, which may be associated with the downregulation of miR-26a expression (10). However, the researchers failed to explore the predictive significance of miR-26a for the disease. In the present study, we confirmed the inhibition of miR-26a expression in PBMCs from 51 newborns with EOS, and newborns with positive hemoculture showed lower levels of miR-26a than those with negative hemoculture. Considering that many factors were unevenly distributed between the groups, we adopted multivariate logistic regression to avoid potential bias and found that the lower expression of miR-26a in PBMCs was independently associated with the occurrence of EOS. This relationship was not affected by the potential confounding factors mentioned above.

Infant and late fetal mortality is often used to assess the national and social protection levels (15). If miR-26a predicts septic death, it may help doctors to take steps in a timely manner, saving lives. In the present study, we found a lower expression of miR-26a in PBMCs in newborns who died than in surviving newborns, but did not find a significant relationship between the miR-26a expression level and the prognosis in the multivariate Cox regression. We believe that the small sample size may be the reason for this negative result.

Indeed, the small sample size is one limitation of this study, although it still achieved statistically significant results. The main reason for the limitation was that there were very few neonatal sepsis cases in our hospital. This same difficulty also affected an international study focusing on miR-26a and neonatal sepsis (10). The solution may be to combine all of the available data from multiple centers into a high-quality meta-analysis.

Furthermore, this study found that the expression of miR-26a in newborns with EOS was significantly higher 72 h after birth than within 72 h of birth. In healthy controls, we did not find such an increasing trend. This information indicates that miR-26a might be involved in the progression of EOS or in some protective mechanisms against EOS. In addition, the present study found a linear relationship between miR-26a and two inflammatory markers (i.e., white blood cell count and C-reactive protein) in newborns with EOS, and thus miR-26a might contribute to some kind of inflammatory or anti-inflammatory process in EOS.

Some studies have found many potential targets of miR-26a, such as Ezh2, PTEN, cyclinD2, cyclinE2, and GSK-3β (11). Other studies have reported that several miRNAs (not miR-26a) can regulate the septic process *via* PTEN/PI3K/AKT signaling in other disease models (14, 16). However, the potential molecular mechanism of miR-26a and PTEN signaling in EOS is not clear. Therefore, the present study focused on this topic and revealed that, with the increase in miR-26a expression from baseline to 72 h after birth, the expressions of PTEN/PI3K/AKT also changed. Moreover, the changes in the expressions of miR-26a and PTEN showed a linear relationship. These findings suggest that PTEN/PI3K/AKT signaling might be involved in the biological effects of miR-26a in newborns with EOS.

However, because some signaling inhibitors that are commonly used in cells and animals cannot be applied to humans, it was difficult to obtain sufficient evidence in order to establish conclusively that miR-26a in EOS was related to inflammation and PTEN/PI3K/AKT signaling. Therefore, we look forward to further *in vivo* and *in vitro* experiments to verify our conclusion.

In addition, previous studies have confirmed the therapeutic effect of miR-26a on obstructive kidney disease and malignant tumors (17–19). Another study focused on tubercle bacillus infection and reported that miR-26a regulates innate immune signaling, macrophage polarization, and the trafficking of *Mycobacterium tuberculosis* to lysosomes (20). As an infectious disease, the main therapy for EOS is antibiotics that target the pathogen. However, the inflammatory response induced by pathogens is an important process during the progression of the disease, and it does not automatically terminate due to pathogen removal (21). An ideal therapeutic strategy for EOS should include antimicrobial and anti-inflammatory therapies. Therefore, given its effect against inflammation, miR-26a has the potential to be a therapeutic target for EOS.

It is well-known that false-negative results of blood culture can easily mislead doctors, resulting in misdiagnosis and failure to treat EOS (22, 23). This phenomenon is usually caused by defects in the blood collection, prior application of antibiotics, irregularities in the culture process, and rare bacterial infections. As a result, doctors usually establish an initial diagnosis based on the clinical symptoms and serological tests and begin empirical therapy. Even if subsequent blood culture findings are negative, doctors generally do not change their original diagnosis and therapeutic plan as long as the symptoms of newborns are gradually ameliorated. In the present study, we confirmed the predictive role of miR-26a in newborns with both positive and negative hemoculture, which could help resolve the deficiencies in blood culture mentioned above.

Compared with EOS, the pathogens, risk factors, and pathogenesis are quite distinct in LOS, which leads to potential differences in the clinical characteristics between the two types of neonatal sepsis (24, 25). Therefore, the predictive role of miR-26a in LOS is unclear and should be fully explored in future research.

In conclusion, our results suggest that a lower baseline expression of miR-26a in PBMCs may identify the occurrence of EOS in Chinese newborns. We also obtained some interesting but not conclusive evidence that the mechanism of miR-26a is related to inflammation and PTEN/PI3K/AKT signaling. More research, including *in vivo* and *in vitro* experiments, should be conducted to more fully explore this topic in the future.

## Data Availability Statement

The datasets presented in this article are not readily available because data not included in the Supplementary File are part of an ongoing study. Requests to access the datasets should be directed to ziyan2317@163.com.

## Ethics Statement

The studies involving human participants were reviewed and approved by the Ethics Committees of Henan Provincial People's Hospital. Written informed consent to participate in this study was provided by the participants' legal guardian/next of kin.

## Author Contributions

ZZ and JZ contributed to the conception and design of the study, collected the data, performed the statistical analysis, wrote, and approved the manuscript. All authors contributed to the article and approved the submitted version.

## Conflict of Interest

The authors declare that the research was conducted in the absence of any commercial or financial relationships that could be construed as a potential conflict of interest.

## Publisher's Note

All claims expressed in this article are solely those of the authors and do not necessarily represent those of their affiliated organizations, or those of the publisher, the editors and the reviewers. Any product that may be evaluated in this article, or claim that may be made by its manufacturer, is not guaranteed or endorsed by the publisher.
